# Morphological Characterization and Pathogenicity Screening of *Fusarium* Isolates Associated with Dry Rot of Stored Potato Tubers in Mascara, Algeria

**DOI:** 10.3390/plants15131999

**Published:** 2026-06-27

**Authors:** Mohamed El Amine Kouadri, Mohammed Chrair, Mohamed Mehenni, Amel Bennacer, Celia Borrero, Manuel Avilés

**Affiliations:** 1 Laboratory of Research on Biological Systems and Geomatics (LRSBG), Department of Agronomy, Faculty of Life and Natural Sciences, University Mustapha Stambouli of Mascara, Mascara 29000, Algeria; 2 Laboratory of Research on Geomatics, Ecology and Environment (LRGEE), University Mustapha Stambouli of Mascara, Mascara 29000, Algeria; chrair.m@univ-mascara.dz; 3 Department of Agronomic Sciences, Faculty of Life and Natural Sciences, University Mustapha Stambouli of Mascara, Mascara 29000, Algeria; 4 Laboratory of Valorization and Conservation of Biological Resources (VALCORE), Department of Biology, Faculty of Sciences, University M’hamed Bougara, Boumerdes 35000, Algeria; a.bennacer@univ-boumerdes.dz; 5 Departamento de Agronomía, ETSIA-Universidad de Sevilla, Ctra. Utrera Km 1, 41013 Sevilla, Spain; cborrero@us.es

**Keywords:** *Fusarium*, potato, dry rot, pathogenicity, TEF1-α, storage diseases, Algeria

## Abstract

Potato (*Solanum tuberosum* L.) tuber dry rot caused by *Fusarium* spp. is a major postharvest disease responsible for significant storage losses worldwide. This study investigated *Fusarium* isolates recovered from symptomatic potato tubers collected in February 2025 from storage facilities in the Mascara region of Algeria. To our knowledge, this is the first regional characterization of *Fusarium*-associated dry rot in stored potato tubers from Mascara, Algeria. By integrating morphological characterization, quantitative pathogenicity assessment, and targeted molecular identification, the study examined the relationship between morphotype occurrence and disease-causing potential. A total of 118 purified *Fusarium* isolates were obtained from symptomatic potato tubers and grouped into 28 morphotypes based on cultural and morphological characteristics. One representative isolate from each morphotype was evaluated for pathogenicity on potato tubers of cv. ‘Arizona’ under controlled incubation conditions, and disease severity was quantified by image analysis. Of the 28 representative isolates tested, 15 induced dry rot symptoms, with marked differences in aggressiveness, whereas several isolates induced little or no necrosis under the conditions tested. Selected aggressive isolates were further analyzed using TEF1-α sequencing, which identified F14 as *F. sambucinum* and F171 as *F. redolens*, while F4 and F34 were identified to the *F. oxysporum* and *F. solani* species-complex level, respectively. Importantly, morphotype frequency did not necessarily reflect disease severity, as some of the most aggressive isolates belonged to less common morphotypes. These findings suggest that isolation frequency alone may not adequately reflect pathogenic importance and support the integration of occurrence data, pathogenicity testing, and molecular identification in the assessment of *Fusarium*-associated dry rot in potato storage systems.

## 1. Introduction

Potato (*Solanum tuberosum* L.) is one of the world’s major staple crops which contributes directly to food security by providing a high-yielding and nutritionally valuable source of carbohydrates, minerals, vitamins, and dietary energy under diverse agro-climatic conditions. However, postharvest losses remain a major constraint on potato production, particularly during storage, where fungal pathogens greatly reduce tuber quality and marketable yield. Among these diseases, dry rot caused by *Fusarium* spp. is one of the most economically damaging, causing major losses in both tuber quantity and quality worldwide [1,2].

The harmfulness of *Fusarium* spp. is not limited to their ability to cause storage diseases and reduce crop yield. Several species of this genus are also important from a food- and feed-safety perspective because they can produce mycotoxins that persist in contaminated plant tissues and may affect humans and animals through the food chain. The most important *Fusarium* mycotoxins include trichothecenes, such as deoxynivalenol (DON), nivalenol (NIV), T-2 toxin, and HT-2 toxin; zearalenone (ZEN); and fumonisins, particularly fumonisin B1 (FB1) [3,4]. These compounds are associated with a wide range of toxic effects, including gastrointestinal disorders, feed refusal, reduced growth and productivity in animals, nephrotoxicity, neurotoxicity, and, in the case of fumonisins, carcinogenic risk [3,5,6]. Therefore, *Fusarium* contamination represents a dual threat: it reduces crop productivity and marketability while also raising concerns for food safety, animal health, and public health.

*Fusarium* species associated with potato dry rot are mainly wound-infecting pathogens that colonize potato tubers during postharvest handling and storage. Disease development is characterized by dry, necrotic lesions that expand internally, leading to tissue collapse, weight loss, and reduced commercial value [7,8]. Several species, including *F. oxysporum*, *F. sambucinum*, *F. solani*, and *F. redolens*, are commonly associated with dry rot, although their prevalence and aggressiveness are strongly influenced by environmental conditions, storage practices, and host genotype [2,9,10].

Studies from different potato-growing regions around the world have shown that potato dry rot is associated with a diverse group of *Fusarium* species, and that the dominant species can vary from one region to another (e.g., *F. coeruleum* in Great Britain, *F. oxysporum* in Michigan, and different species complexes in Poland and Russia) [1,11,12]. These studies have also shown that pathogenic aggressiveness differs not only between species, but also between isolates of the same species [10,11]. This suggests that isolation frequency does not necessarily reflect disease importance, since frequently isolated taxa may be weakly pathogenic or even non-pathogenic under tuber assay conditions [11,12]. In addition, previous work has highlighted the importance of combining molecular identification with pathogenicity tests to better understand the role of *Fusarium* populations in storage environments [1,13]. Overall, these findings show that local studies are important, since the composition and aggressiveness of *Fusarium* populations are influenced by regional production and storage conditions.

Recent research indicates that the diversity of *Fusarium* species associated with potato dry rot may be greater than previously assumed and can differ substantially across production regions. In the Pacific Northwest of the United States, molecular phylogenetic analysis of 327 isolates revealed 20 *Fusarium* species, 14 of which were confirmed to be pathogenic on potato tubers. Likewise, a recent multilocus study conducted in Shanxi Province, China, identified five pathogenic species and reported *F. dimerum* as part of the local dry rot complex [14,15]. Together with recent findings from India and Kyrgyzstan, these studies highlight the strong influence of geography, storage conditions, and host background on the structure of local *Fusarium* populations. They also underscore the importance of region-specific surveys that integrate molecular identification with pathogenicity testing [13,16].

In Algeria, potato production is of major socio-economic importance. Mascara is one of the main potato-growing and storage areas in northwestern Algeria. Mascara lies within the Ghriss Plain, a fertile agricultural basin known for intensive potato and onion production [17]. Because large quantities of tubers are produced and stored in Mascara, this region is well suited for studying storage-associated dry rot under conditions typical of potato production in western Algeria. The semi-arid climate, combined with inadequate storage management, especially poor control of temperature, humidity, and ventilation, creates a favorable environment for the development of storage pathogens, especially *Fusarium* spp. [18,19].

A major challenge in understanding *Fusarium* dry rot is the taxonomic complexity and functional diversity within the genus. Morphological identification is often unreliable because many species share similar phenotypic traits, while isolates within the same species can exhibit substantial differences in aggressiveness [20]. These differences are associated with variation in genetic background, secondary metabolite production (including mycotoxins), and the ability to produce enzymes that degrade host tissue [16,21].

Molecular tools, particularly sequencing of the translation elongation factor 1-alpha (TEF1-α) gene, have significantly improved species-level identification and phylogenetic resolution within *Fusarium* [22]. In parallel, disease severity is often assessed visually, but digital image analysis can provide more objective and reproducible comparisons between isolates [23,24,25]. Recent experimental studies also emphasize the importance of integrating pathogenicity assays with quantitative evaluation to better discriminate aggressiveness among *Fusarium* isolates under controlled conditions [14,26,27].

Previous Algerian studies on potato tuber dry rot have addressed related but complementary questions. Azil et al. [9] surveyed *Fusarium* associated with both potato wilt and tuber dry rot across several provinces and assessed pathogenicity using a broader molecular framework, whereas Benhaoued et al. [28] reported a broader range of postharvest fungal pathogens in El Oued, while Hadjebar et al. [29] focused on mycotoxin occurrence and toxigenic potential of dry-rot-associated *Fusarium* isolates from Bouira and Ain Defla.

Although *Fusarium* has been reported in potato diseases in Algeria, the pathogenic variability of isolates recovered from storage remains poorly characterized. In addition, molecular identification has rarely been directly linked with quantitative aggressiveness data in the same study. For the Mascara region, information remains limited, and no previous study has characterized *Fusarium*-associated potato dry rot in stored tubers from this area. This knowledge gap includes uncertainty regarding which isolates are most relevant in stored tubers and whether isolate occurrence is associated with pathogenic aggressiveness.

Accordingly, this study aimed to recover *Fusarium* isolates from potato tubers showing dry-rot symptoms in Mascara, characterize them using morphological traits, evaluate the pathogenicity and aggressiveness of representative morphotype isolates on potato cv. ‘Arizona’, and support the identification of selected aggressive isolates using TEF1-α sequencing. By linking morphological characterization with pathogenicity data, this work provides a regional assessment of *Fusarium*-associated dry rot in Algerian potato storage systems.

## 2. Results

### 2.1. Sampling and Symptom Description

**Figure 1:**
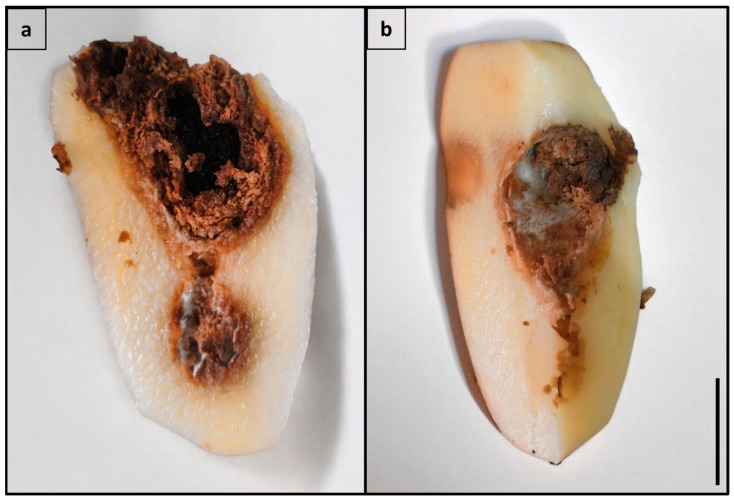
Symptoms of potato tuber dry rot observed in sampled storage tubers. (**a**) Tubers at an advanced stage of infection. (**b**) Tubers at an intermediate stage. Scale bar = 3 cm.

After sampling and transfer to the laboratory, tubers were carefully sectioned through the center to observe and confirm symptoms consistent with *Fusarium* dry rot. Some tubers were already at an advanced stage of infection, showing extensive tissue breakdown; the affected areas were dry, friable, and dark brown to black. These tubers typically exhibited a wrinkled periderm and marked internal tissue degradation (Figure 1a).

In contrast, other tubers were at an intermediate stage, characterized by localized discoloration (generally brown to yellow) and firmer tissues. Although symptom expression was clear, tissue degradation was less severe than in advanced infections, and the tubers remained partly firm despite changes in texture (Figure 1b).

### 2.2. Isolation Frequency and Morphological Characterization of Fusarium Isolates

Isolation from symptomatic potato tubers yielded 118 purified isolates showing cultural and microscopic features consistent with the genus *Fusarium*. Based on colony morphology and microscopic characters, the isolates were separated into 28 morphotypes. The most represented morphotypes were F3, F2, and F56, accounting for 11.86%, 10.17%, and 6.78% of the isolates, respectively, whereas the remaining morphotypes occurred at lower frequencies ranging from 0.85% to 6.78% (Table 1). One representative isolate from each morphotype was selected for pathogenicity testing, and selected aggressive isolates were further characterized by TEF1-α sequencing.

Macroscopic examination of the *Fusarium* isolates revealed marked variability in colony morphology, particularly in the development of aerial mycelium and colony pigmentation. Representative colony morphology and microscopic observations of all 28 isolates are provided in Supplementary Figure S1. These observations were used to document phenotypic variability within the recovered population and to support the selection of representative isolates for pathogenicity screening.

Differences in colony texture were evident, ranging from abundant aerial growth with floccose mycelium (F15) to cottony colonies (F67), velvety colonies with moderate aerial hyphae (F4, F6, F7), woolly colonies with dense, raised mycelium (F52, F54), and glabrous/flat colonies with sparse aerial mycelium (F2, F3).

Colony pigmentation also showed wide diversity, with surface colors ranging from white (F56) to yellow (F11), as well as pink (F54) and violet–mauve (F7, F21). Distinct pigmentation patterns were noted in some types, including a red central zone (F2, F6), central brown pigmentation (F96), and yellow spots at the colony periphery (F3).

### 2.3. Pathogenicity and Aggressiveness of Representative Fusarium Isolates

**Figure 2:**
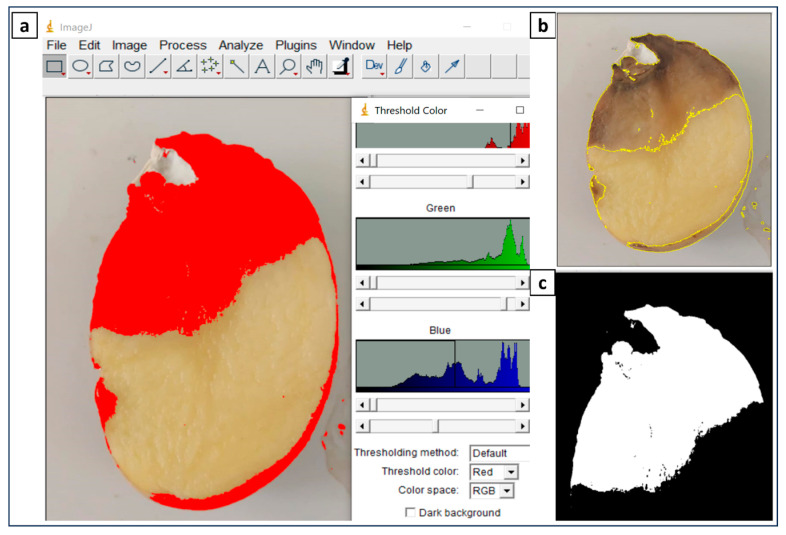
Image-based quantification of tuber dry rot severity using ImageJ. Representative cross-section of an infected potato tuber used to measure diseased tissue area relative to total tuber cross-section. The same color-threshold settings were applied to all images before measurement. (**a**) color thresholding, (**b**) area selection, (**c**) binary mask generation for the infected area.

**Figure 3:**
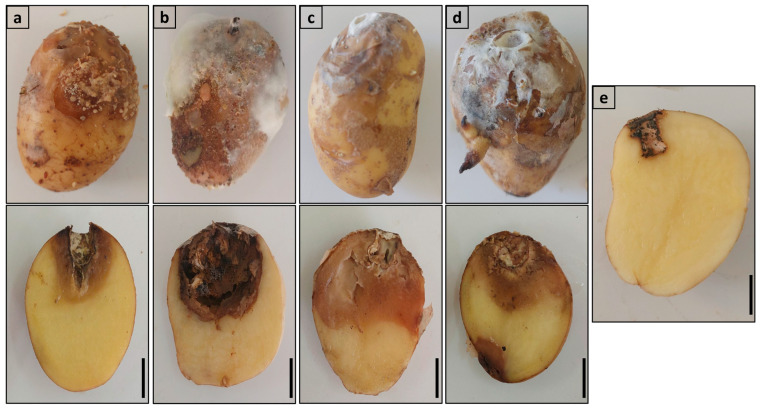
External and internal symptoms of *Fusarium* dry rot produced during the pathogenicity test on potato tubers, (**a**) *F. oxysporum* species complex (F4), (**b**) *F. sambucinum* (F14), (**c**) *F. solani* species complex (F34), (**d**) *F. redolens* (F171), (**e**) control. Scale bar = 2 cm.

**Figure 4:**
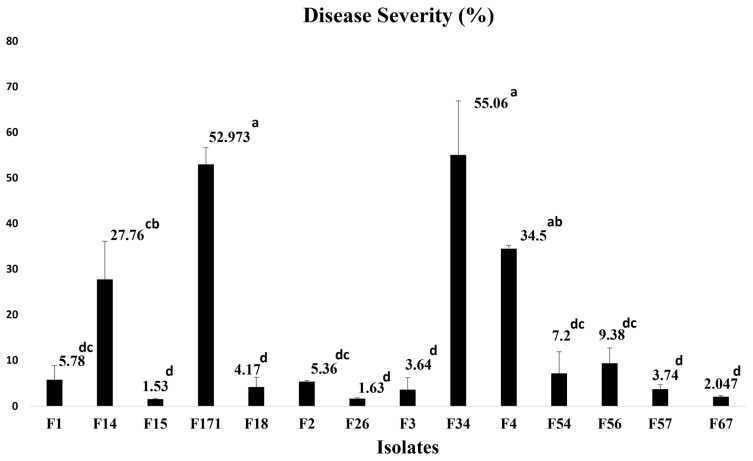
Disease severity caused by representative isolates of *Fusarium* in the tuber pathogenicity assay on potato cv. ‘Arizona’. Bars represent the mean of three independent run means (five tubers per run); error bars indicate the standard error among runs. Different letters above bars indicate significant differences among isolates according to Tukey’s HSD test at *p* ≤ 0.05. Raw disease-severity values used for statistical analysis are provided in Supplementary Table S1.

Disease severity was quantified from tuber cross-sections by ImageJ analysis, allowing objective comparison of symptom development among representative isolates (Figure 2). Based on the pathogenicity assay, 15 of the 28 tested representatives induced dry rot symptoms on cv. ‘Arizona’, with clear differences in aggressiveness (Figure 3 and Figure 4).

#### 2.3.1. Image-Based Quantification of Tuber Rot Severity

Rot severity was assessed from cross-sectional images using ImageJ (Figure 2). The most pathogenic representatives produced extensive necrotic areas, whereas weakly pathogenic ones caused only limited discoloration and necrosis.

#### 2.3.2. Aggressiveness and Symptom Expression of *Fusarium* Isolates

Isolates F34 and F171 showed the highest mean disease severity values, 55.06% and 52.97%, respectively, and did not differ significantly from each other according to Tukey’s HSD test (*p* ≤ 0.05). Isolate F4, with a mean disease severity of 34.50%, was also not significantly different from F34 or F171. These isolates produced typical internal and external dry rot symptoms (Figure 3). Internally, infected tubers developed dry, necrotic lesions ranging from dark brown to black, often extending deeply into the tuber and leading to hollow cavities and areas of desiccated tissue, consistent with advanced dry rot.

Externally, tubers exhibited sunken, dark, wrinkled areas, usually centered around the inoculation site, and visible white *Fusarium* mycelium was observed on the tuber surface. Isolate F14 showed a mean disease severity of 27.77% and did not differ significantly from F4, but was significantly less aggressive than F34 and F171. In contrast, F15 (1.53%), F26 (1.63%), and F67 (2.04%) showed low pathogenicity and produced only small lesions (Figure 4). Control tubers showed only slight wound-associated discoloration and did not develop typical dry rot symptoms.

### 2.4. Molecular Identification

Based on the pathogenicity results, the four most aggressive representative isolates were selected for TEF1-α sequencing. BLASTn (version 2.16.0) analysis of the obtained TEF1-α sequences showed that F14 and F171 matched *Fusarium sambucinum* strain MFG 60833 (OR020701.1) and *Fusarium redolens* isolate GR_FR29 (MT305219.1), respectively, with 100% nucleotide identity and 100% query cover. Isolate F4 showed 100% identity and 100% query cover with *Fusarium oxysporum* isolate FS11476a (MN417194.1), supporting its placement within the *F. oxysporum* species complex, whereas F34 showed 100% identity and 100% query cover with *Fusarium solani* isolate F80 (OQ511045.1), supporting its placement within the *F. solani* species complex (Table 2). Based on these results, the selected aggressive isolates were identified as *F. sambucinum* (F14), *F. redolens* (F171), *F. oxysporum* species complex (F4), and *F. solani* species complex (F34). The TEF1-α sequences obtained in this study were submitted to GenBank under accession numbers PX985284.1 (F4), PX985286.1 (F14), PX985283.1 (F34), and PX985285.1 (F171).

**Figure 5:**
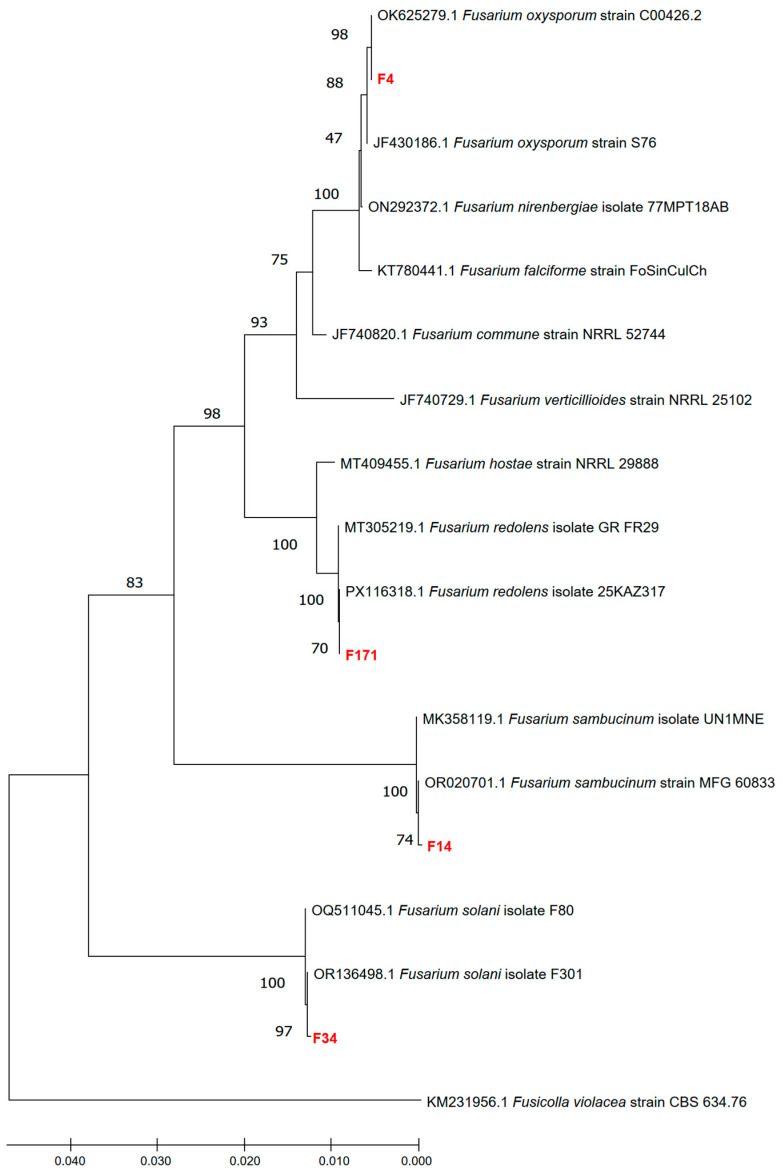
Neighbour-joining phylogenetic tree of the selected aggressive *Fusarium* isolates based on TEF1-α sequences. Isolates obtained in this study are shown in red. Closely related reference sequences were retrieved from GenBank. Bootstrap values based on 1000 replicates are indicated at the branches. *Fusicolla violacea* was used as the outgroup.

Phylogenetic analysis was performed using the TEF1-α sequences of the selected aggressive isolates together with closely related *Fusarium* reference sequences retrieved from GenBank (Figure 5). Isolates F14 and F171 were grouped with reference sequences of *F. sambucinum* and *F. redolens*, respectively, whereas F4 and F34 clustered within the *F. oxysporum* and *F. solani* species complexes. These results support the TEF1-α-based identification of the selected aggressive isolates and confirm their placement within distinct *Fusarium* lineages associated with potato dry rot.

## 3. Discussion

This study provides a regional characterization of *Fusarium* isolates associated with potato dry rot in storage facilities in the Mascara region, based on morphological traits, pathogenicity assays, and TEF1-α-based identification of selected aggressive isolates. The findings confirm that *Fusarium* spp. are associated with postharvest dry rot in the sampled storage facilities, in agreement with previous studies identifying this genus as a major cause of storage losses in potato systems worldwide [1,2,30]. The morphological and pathogenic variability observed among the recovered isolates supports previous reports that potato dry rot is associated with diverse *Fusarium* populations rather than a single pathogen [31].

Species such as *Fusarium sambucinum*, *Fusarium oxysporum*, *Fusarium solani*, and *Fusarium redolens* are commonly reported in association with this disease, although their relative prevalence and pathogenicity may vary depending on environmental conditions, storage practices, and host [2,12]. In particular, *F. sambucinum* is frequently described as one of the most aggressive species responsible for severe dry rot symptoms, whereas *F. oxysporum* is frequently isolated from infected tubers but is generally less aggressive and more variable in tuber pathogenicity assays [14,32].

The selected aggressive isolates identified in this study are partly consistent with earlier Algerian reports, and provide additional evidence of pathogenic variability among *Fusarium* isolates associated with potato dry rot in Mascara. Azil et al. [9], working across multiple Algerian provinces on both wilt and dry rot, identified *F. sambucinum* as the dominant species associated with dry rot and the most aggressive taxon in tuber assays. Hadjebar et al. [29] also detected dry-rot-associated isolates belonging to the *F. sambucinum*, *F. oxysporum*, *F. redolens*, *F. tricinctum*, and *F. incarnatum-equiseti* species complexes in Bouira and Ain Defla, although their study focused on mycotoxin occurrence rather than quantitative pathogenicity. By contrast, Benhaoued et al. [28] reported *F. proliferatum* together with non-*Fusarium* postharvest pathogens in El Oued, without aggressiveness testing. In comparison with previous Algerian studies, the present results show that the aggressive isolates from Mascara included taxa with different levels of aggressiveness under the assay conditions. These findings fill an important regional knowledge gap for Mascara where data on *Fusarium*-associated potato dry rot in stored tubers remain limited.

The marked variation in colony morphology and pigmentation among the isolates reflects the well-known phenotypic plasticity of *Fusarium* spp. Differences in mycelial texture and color are often associated with underlying genetic diversity and adaptive responses to environmental conditions [33,34,35,36,37]. In particular, pigmentation has been linked to the biosynthesis of secondary metabolites such as naphthoquinones and fusarubins, which are known to contribute to fungal development, stress tolerance, and pathogenicity [21,33]. These traits may contribute to the ecological fitness of *Fusarium* isolates in storage environments with fluctuating temperature and humidity, as observed in the Mascara region.

A key finding of this study is the large variation in pathogenicity among isolates. Selected isolates from morphotypes F34 and F171 induced extensive tissue maceration and greater disease severity, whereas others, including selected isolates from the types F15, F26, and F67, caused little or no disease. Such variability has been widely reported in *Fusarium* populations infecting potato and reflects differences in virulence factors, including the production of cell wall-degrading enzymes (e.g., pectinases and cellulases), phytotoxic compounds, and other metabolites involved in host colonization [1,38].

Highly aggressive isolates of *Fusarium* are generally more effective at colonizing potato tuber tissues and causing larger lesions, whereas weakly pathogenic isolates may show reduced virulence-related activity, lower compatibility with a given host cultivar, or stronger dependence on favorable environmental and storage conditions for disease expression [2,10,39].

The high aggressiveness of F171 (*F. redolens*) and F34 (*F. solani* species complex) is notable, as previous Algerian studies mainly identified *F. sambucinum* as the most aggressive dry-rot taxon and described *F. redolens* as an associated member of the *Fusarium* complex rather than a major tuber-rot isolate [9,29]. By contrast, our results showed that F171 and F34 were highly aggressive, whereas F14 *(F. sambucinum)* exhibited only moderate aggressiveness. These results suggest that dry rot risk in Mascara storage systems may also be driven by less frequently emphasized *Fusarium* taxa with high pathogenic potential.

Conversely, the intermediate aggressiveness observed for F4 (*F. oxysporum* species complex) in the present study is broadly consistent with Azil et al. [9], who recovered *F. oxysporum* from Algerian potato material but did not find their tuber-tested isolates to be strongly pathogenic; differences in cultivar, incubation temperature, and assay design between the two studies may partly explain this contrast. The use of TEF1-α sequencing provided molecular support for the identification of the selected aggressive isolates [22,40,41,42].

Beyond species identification, differences in aggressiveness among the Mascara isolates can be interpreted within the framework of host–pathogen interactions. Because *Fusarium* dry rot is mainly a wound-mediated disease, variation in aggressiveness may reflect differences in the ability of isolates to colonize wounded tissues, overcome wound-healing barriers, and adapt to cultivar-specific characteristics [2,14,16]. Previous studies have also shown that cultivar resistance can strongly influence lesion development and disease severity [10]. This factor should be considered in the present study because cv. ‘Arizona’ is classified as moderately resistant to *Fusarium* dry rot [43]. This resistance may have reduced symptom development, particularly for isolates with low aggressiveness. Therefore, the results represent the relative aggressiveness of the tested isolates on cv. ‘Arizona’ and may differ on a more susceptible cultivar. The inclusion of a highly susceptible cultivar could have provided clearer differentiation among isolates and should be considered in future studies.

Storage ecology is likewise critical, because temperature, relative humidity, and storage duration can influence fungal growth, lesion expansion, and mycotoxin accumulation, thereby affecting which *Fusarium* taxa become most damaging under storage conditions [2,18]. Therefore, isolates showing weak or no pathogenicity under the present assay conditions may still have ecological relevance under different storage environments or host conditions.

An important result of the pathogenicity assay is that the frequency of isolation of a morphotype did not necessarily reflect its aggressiveness on potato tubers. Some frequently recovered isolates were pathogenic, whereas others produced only limited necrosis or failed to cause typical dry rot symptoms under the assay conditions. This was observed for F34 and F171, which caused the highest disease severity although they were not among the most frequent morphotypes in the collection. Similar differences between isolation frequency and aggressiveness have been reported in potato dry rot studies based on species-level identification; for example, in Michigan, *Fusarium oxysporum* was the most commonly recovered species, whereas *F. sambucinum* was the most aggressive [11]. A similar pattern was observed in Poland, where *F. oxysporum* was the most frequently recovered species, although pathogenic isolates were primarily identified as *F. sambucinum*, *F. avenaceum*, *F. culmorum*, and *F. graminearum* [12]. This highlights the importance of distinguishing between pathogen presence and disease-causing potential when assessing dry rot risk in storage systems.

From a disease-management perspective, the presence of highly aggressive isolates at low isolation frequency is important. If attention is given only to the most frequently recovered morphotypes, isolates with strong pathogenic potential may be overlooked. Such isolates may still contribute to storage losses when they colonize wounded tuber tissues under favorable storage conditions [1,2,14]. Therefore, dry rot monitoring should combine isolation frequency with pathogenicity testing, and, where possible, molecular identification of isolates showing high disease potential [1,14,16].

This study represents an initial characterization of *Fusarium* isolates associated with potato dry rot in stored tubers from Mascara, an important potato production and storage region in western Algeria. However, the findings should be interpreted within the limits of the experimental design. Sampling was restricted to symptomatic tubers collected from six storage facilities during a single month, and pathogenicity was assessed using one potato cultivar (‘Arizona’) under one controlled incubation regime. Therefore, the present results provide a regional baseline for the sampled storage context, but they do not capture possible seasonal variation, differences among potato cultivars, or the influence of alternative storage environments on disease expression and isolate aggressiveness. In addition, molecular identification was carried out only for four highly aggressive isolates, and post-inoculation re-isolation was confirmed only at the genus level based on colony and microscopic traits consistent with *Fusarium*. Further work should therefore include broader sampling across seasons and locations, assessment on additional potato cultivars, evaluation under different storage conditions, molecular characterization of a larger number of isolates, and more precise confirmation of re-isolated fungi after pathogenicity assays.

These findings indicate that potato storage systems in Mascara would benefit from an integrated disease management strategy. Since *Fusarium* dry rot is a wound-mediated disease, the first priority should be to minimize mechanical injury during harvesting, transport, grading, and storage. This effort should be complemented by rigorous sanitation of storage facilities, the systematic removal of infected tubers, and better control of storage temperature, humidity, and ventilation [2,18,30].

In addition to conventional storage management, future work should evaluate biological and botanical control options under local conditions. Recent studies suggest that microbial antagonists such as *Trichoderma asperellum* and plant-derived antifungal products may inhibit *Fusarium* growth and reduce potato dry rot, but their effectiveness should be validated against locally aggressive isolates [44,45,46,47].

Overall, effective management of *Fusarium*-associated dry rot in Mascara should combine surveillance of highly aggressive isolates with practical measures that reduce infection opportunities, including wound prevention, storage sanitation, environmental control, and cultivar susceptibility screening.

## 4. Materials and Methods

### 4.1. Sampling and Isolation

**Figure 6:**
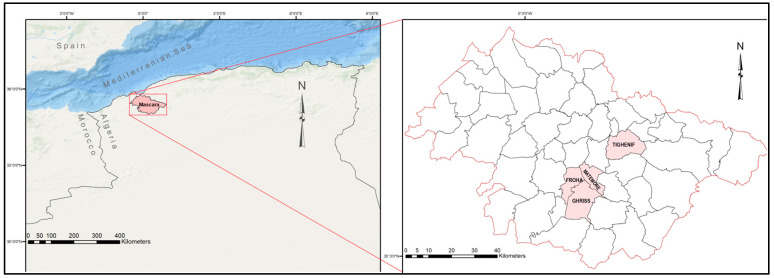
Geographic location of the study area and sampling sites in the Mascara region, Algeria.

A total of 100 symptomatic potato cv. ‘Arizona’ tubers were collected during February 2025 from 6 storage facilities in four regions of Mascara (Froha, Matemour, Ghriss and Tighnif), with 15–20 tubers sampled from each facility (Figure 6).

The surveyed facilities differed in storage management, but in general tubers were maintained under commercial storage conditions typical of the Mascara region, where temperature, relative humidity, and ventilation were not strictly controlled and likely varied among facilities. Tubers showing symptoms consistent with dry rot were randomly selected within each facility, placed in Kraft paper bags, and transported immediately to the laboratory for fungal isolation. Tissue fragments were taken from the lesion margins of each tuber to isolate actively growing fungi.

Fungal isolation was performed after surface disinfection of the tubers using 2% sodium hypochlorite for 3 min, followed by thorough rinsing in sterile distilled water for 3 min. After drying with sterile filter paper, tubers were bisected, and small sections (0.3–0.5 cm) of symptomatic tissue were aseptically excised from the margins of lesions using a sterile scalpel. The fragments were plated onto potato dextrose agar (PDA; 200 g potato, 20 g dextrose, and 15 g agar/L) in Petri dishes (5–6 fragments per plate). Plates were incubated at 25 °C for 5–6 days.

Fungal colonies showing *Fusarium*-like morphology were purified by hyphal-tip transfer onto fresh PDA. Additional purification was performed from conidial suspensions plated on PDA to reduce mixed cultures. When more than one *Fusarium*-like colony was obtained from the same tuber, isolates with similar cultural and morphological features were assigned to the same morphotype, whereas clearly distinct isolates were assigned to separate morphotypes. One representative isolate from each morphotype was retained for morphological observation and pathogenicity testing.

*Fusarium* morphotype frequency (%) was calculated by dividing the number of isolates assigned to each morphotype by the total number of *Fusarium* isolates obtained from symptomatic tubers and multiplying by 100.

### 4.2. Morphological Characterization

Morphological characterization was conducted to describe phenotypic variability among morphotype groups and to support the selection of representative isolates for pathogenicity screening. Isolates were examined for cultural and morphological features consistent with *Fusarium*, including colony appearance and microscopic characters such as hyaline septate hyphae, fusiform to falcate macroconidia with several septa, microconidia in some isolates, and the occurrence of chlamydospores in culture, following the criteria of Leslie and Summerell [48]. Morphology alone was not sufficient for definitive species identification. Although individual morphological traits varied among the representative isolates, all 28 morphotypes displayed morphological features consistent with the genus *Fusarium*.

### 4.3. Pathogenicity Assay

Pathogenicity was evaluated using one representative isolate from each *Fusarium* morphotype by inoculating potato tubers and incubating them under controlled conditions at 25 °C [1]. Healthy tubers of the cultivar ‘Arizona’ were selected because this cultivar is widely grown and stored in Algeria, making it representative of the local production and storage system, and because it is widespread in the Mediterranean area and highly adaptable to early cultivation [49]. Tubers were surface-disinfested in 1% sodium hypochlorite for 5 min, rinsed with sterile distilled water, and air-dried.

Tubers were then wounded using a 5 mm cork borer to a depth of 1 cm. Agar plugs (4 mm in diameter) were excised from 7-day-old fungal cultures grown on potato dextrose agar (PDA) and inserted into the wounds with the mycelium-covered side facing the tuber tissue; the cavities were subsequently sealed with the previously removed tuber tissue [13]. Control tubers received sterile PDA plugs without fungal mycelium. Control tubers were included in each experimental run and incubated under the same conditions as inoculated tubers. All tubers were placed in plastic boxes (8.5 cm × 19 cm × 31 cm), loosely covered to allow gas exchange, and incubated at 25 °C for 4 weeks in the dark. After incubation, tubers were cut through the inoculation site to evaluate internal symptom development. Each run included five tubers per isolate, and the experiment was repeated three times, resulting in 15 tuber observations per isolate across all runs.

In this study, pathogenicity was defined as the ability of an isolate to induce dry rot symptoms after inoculation, whereas aggressiveness was defined as the relative severity of disease caused by pathogenic isolates. Disease severity was expressed as the percentage of necrotic tuber cross-sectional area.

### 4.4. Disease Assessment

Digital images of tuber cross-sections were analyzed using ImageJ v1.54 (National Institutes of Health, USA). To ensure comparability among samples, all cross-sections were photographed under standardized conditions, with the camera positioned at a fixed distance from the sample, under uniform artificial lighting, and against a plain background. Image analysis was performed blind to isolate identity by coding the tuber images before measurement. Images were imported into ImageJ, and necrotic tissue was segmented from healthy tissue using the Color Threshold function. The same threshold settings were applied to all tuber images to ensure consistency across samples. Images were processed using the same workflow for all samples, including threshold application, binary mask generation, and area measurement. The segmented diseased area was converted into a binary mask and measured, and the total cross-sectional area of each tuber was also determined. Disease severity was then calculated as the percentage of necrotic area relative to the total tuber cross-sectional area. For each tuber, disease severity was calculated as:

Disease severity (%) = (necrotic area/total tuber cross-sectional area) × 100

To support Koch’s postulates at the genus level, fungi were re-isolated from the margins of symptomatic tissue developing around the inoculation site. The recovered fungi showed colony and microscopic characteristics consistent with *Fusarium*, confirming association of the inoculated *Fusarium* cultures with the observed dry rot symptoms at the genus level.

### 4.5. TEF1-α-Based Identification of Selected Aggressive Isolates

For TEF1-α-based identification, four representative *Fusarium* isolates showing the highest aggressiveness in the pathogenicity test were selected for genomic DNA extraction from fresh mycelium. These isolates were subjected to PCR amplification using the primer pair EF1/EF2, and the amplified products were analyzed by Sanger dideoxy sequencing. Each PCR reaction had a final volume of 24 µL and contained 1 µL of DNA template, 14.75 µL of nuclease-free water, 5 µL of 5× Q5 Reaction Buffer, 0.5 µL of 10 mM dNTP mix, 1.25 µL of primer EF1 (10 µM), 1.25 µL of primer EF2 (10 µM), and 0.25 µL of Q5 High-Fidelity DNA Polymerase (5 U/µL). The primer sequences used were EF1: 5′-ATGGGTAAGGARGACAAGAC-3′ and EF2: 5′-GGARGTACCAGTSATCATG-3′ [50].

PCR amplification was performed with an initial denaturation at 98 °C for 30 s, followed by 35 cycles of denaturation at 98 °C for 10 s, annealing at 55 °C for 30 s, and extension at 72 °C for 20 s, with a final extension at 72 °C for 2 min. Amplicons were resolved by agarose gel electrophoresis using a 100 bp DNA ladder, and the resulting PCR products were subsequently subjected to Sanger dideoxy sequencing for identification of the selected aggressive *Fusarium* isolates.

The obtained sequences were analyzed using BLASTn against the NCBI GenBank nucleotide database. For each selected isolate, the closest BLAST hit was recorded together with the corresponding accession number, query length, percentage identity, query cover, maximum score, and E-value. Taxonomic identification was based on the closest reference matches, sequence similarity, and phylogenetic placement.

For phylogenetic analysis, the sequences generated in this study were aligned with closely related *Fusarium* reference sequences retrieved from GenBank using MUSCLE. A neighbour-joining phylogenetic tree was constructed in MEGA X (version 10.2.6) [51] with 1000 bootstrap replicates, and *Fusicolla violacea* was used as the outgroup. The analytical procedure encompassed 18 nucleotide sequences and 968 positions.

### 4.6. Statistical Analysis

All statistical analyses were performed using JASP software (v0.19.3; JASP Team, Amsterdam, The Netherlands). For each isolate, disease severity was first calculated for each individual tuber. The five tubers within each experimental run were then averaged to obtain one run mean per isolate. These run means (*n* = 3 per isolate) were used as the experimental units for statistical analysis. Prior to ANOVA, model assumptions were assessed by testing residual normality and homogeneity of variance using the Shapiro–Wilk and Levene’s tests, respectively. Based on these diagnostic checks, data transformation was not considered necessary. Disease severity data were analyzed by one-way analysis of variance (ANOVA), and mean separation was conducted using Tukey’s honestly significant difference (HSD) post hoc test at *p* ≤ 0.05. Different letters indicate statistically significant differences among isolates.

## 5. Conclusions

This study provides the first regional overview of the morphological variability and pathogenic potential of *Fusarium* isolates associated with potato tuber dry rot in storage facilities in Mascara, Algeria.

Pathogenicity tests using one representative isolate from each of the 28 morphotypes showed that only some representative isolates induced typical dry rot symptoms, and that aggressiveness varied considerably among isolates. TEF1-α sequencing of selected aggressive isolates identified F14 as *F. sambucinum* and F171 as *F. redolens*, while F4 and F34 were identified to the *F. oxysporum* and *F. solani* species-complex level, respectively.

The results showed that morphotype frequency and morphological characteristics were not clear indicators of aggressiveness. Some less common morphotypes included highly aggressive isolates, whereas some frequently recovered morphotypes showed low disease severity. This suggests that surveys based only on morphology or isolation frequency may not identify the isolates that pose the greatest risk during storage. A more accurate assessment of *Fusarium* dry rot should therefore combine occurrence data with pathogenicity testing and molecular identification of aggressive isolates.

Overall, this work contributes to a better understanding of *Fusarium*-associated dry rot in Algerian potato storage systems and provides a useful basis for future studies involving broader molecular characterization of the recovered isolates. For disease monitoring, attention should not be limited to the most frequently isolated morphotypes, because less common morphotypes may also include highly aggressive isolates.

In practical terms, farmers and storage operators should focus on reducing tuber wounding, removing symptomatic tubers, and improving hygiene and storage conditions to limit disease development. Future studies should examine a broader range of isolates, cultivars, and storage environments to determine whether the same pattern occurs in other potato-growing regions.

## Figures and Tables

**Table 1:** Isolation frequency of *Fusarium* morphotypes obtained from symptomatic potato tubers.

Morphotype	Frequency (%)	Morphotype	Frequency (%)	Morphotype	Frequency (%)
F1	1.69	F17	4.24	F54	1.69
F2	10.17	F18	2.54	F56	6.78
F3	11.86	F21	0.85	F57	1.69
F4	2.54	F23	0.85	F59	3.39
F6	6.78	F26	2.54	F60	1.69
F7	0.85	F33	6.78	F67	4.24
F11	2.54	F34	3.39	F81	2.54
F14	3.39	F40	4.24	F96	2.54
F15	3.39	F52	3.39	F103	0.85
—	—	—	—	F117	2.54

Frequency (%) was calculated as the proportion of purified *Fusarium* isolates assigned to each morphotype relative to the total number of isolates recovered from symptomatic tubers (n = 118).

**Table 2:** Molecular identification of the selected aggressive *Fusarium* isolates based on TEF1-alpha sequence analysis using BLASTn. The table shows the query accession number, closest BLAST hit, hit accession number, query length, percentage identity, query cover, maximum score, and E-value.

Isolate Code	Query Accession	Closest BLAST Hit (Species/Strain)	Hit Accession	Query Length (bp)	Identity (%)	Query Cover (%)	Max Score	E-Value
F4	PX985284.1	*Fusarium oxysporum* isolate FS11476a	MN417194.1	654	100.00	100	1208	0.0
F14	PX985286.1	*Fusarium sambucinum* strain MFG 60833	OR020701.1	633	100.00	100	1170	0.0
F34	PX985283.1	*Fusarium solani* isolate F80	OQ511045.1	710	100.00	100	1312	0.0
F171	PX985285.1	*Fusarium redolens* isolate GR_FR29	MT305219.1	684	100.00	100	1264	0.0

## Data Availability

All data are contained within this article and the Supplementary Materials.

## Supplementary Materials

The following supporting information can be downloaded at: https://www.mdpi.com/article/10.3390/plants15131999/s1, Figure S1: Macroscopic and microscopic observations of selected isolates in this study; Table S1: Disease-severity values of representative *Fusarium* isolates used for statistical analysis.

## Abbreviations

The following abbreviations are used in this manuscript:

ANOVA	Analysis of Variance
BLASTn	Basic Local Alignment Search Tool for nucleotide sequences
bp	base pair(s)
cv.	cultivar
DNA	Deoxyribonucleic Acid
dNTP	Deoxynucleotide Triphosphate
EF1/EF2	Elongation Factor 1 primer pair
GenBank	Genetic Sequence Database of the National Center for Biotechnology Information
HSD	Honestly Significant Difference
ImageJ	Image Processing Program developed at the National Institutes of Health
NCBI	National Center for Biotechnology Information
PDA	Potato Dextrose Agar
PCR	Polymerase Chain Reaction
TEF1-α	Translation Elongation Factor 1-alpha
µL	microliter
µM	micromolar
°C	degree Celsius

## References

[1] Gavrilova O., Orina A., Trubin I., Gagkaeva T. (2024). Identification and pathogenicity of *Fusarium* fungi associated with dry rot of potato tubers. Microorganisms.

[2] Xue H., Liu Q., Yang Z. (2023). Pathogenicity, mycotoxin production, and control of potato dry rot caused by *Fusarium* spp.: A review. J. Fungi.

[3] Qu Z., Ren X., Du Z., Hou J., Li Y., Yao Y., An Y. (2024). *Fusarium* mycotoxins: The major food contaminants. mLife.

[4] Pinton P., Terciolo C., Payros D., Oswald I.P. (2025). Mycotoxins hazard: The European view. Curr. Opin. Food Sci..

[5] Khan R., Anwar F., Mohamad Ghazali F. (2024). A comprehensive review of mycotoxins: Toxicology, detection, and effective mitigation approaches. Heliyon.

[6] Yilmaz N., Verheecke-Vaessen C., Ezekiel C.N. (2025). Mycotoxins: An ongoing challenge to food safety and security. PLoS Pathog..

[7] Tiwari R.K., Bashyal B.M., Shanmugam V., Lal M.K., Kumar R., Sharma S., Vinod, Gaikwad K., Singh B., Aggarwal R. (2021). Impact of *Fusarium* dry rot on physicochemical attributes of potato tubers during postharvest storage. Postharvest Biol. Technol..

[8] Tiwari R.K., Lal M.K., Kumar R., Sharma S., Sagar V., Kumar A., Singh B., Aggarwal R. (2023). Impact of *Fusarium* infection on potato quality, starch digestibility, in vitro glycemic response, and resistant starch content. J. Fungi.

[9] Azil N., Stefańczyk E., Sobkowiak S., Chihat S., Boureghda H., Śliwka J. (2021). Identification and pathogenicity of *Fusarium* spp. associated with tuber dry rot and wilt of potato in Algeria. Eur. J. Plant Pathol..

[10] Christian C.L., Duellman K.M. (2026). Relative aggressiveness of *Fusarium* dry rot pathogens on seven potato varieties. Am. J. Potato Res..

[11] Gachango E., Hanson L.E., Rojas A., Hao J.J., Kirk W.W. (2012). *Fusarium* spp. causing dry rot of seed potato tubers in Michigan and their sensitivity to fungicides. Plant Dis..

[12] Stefańczyk E., Sobkowiak S., Brylińska M., Śliwka J. (2016). Diversity of *Fusarium* spp. associated with dry rot of potato tubers in Poland. Eur. J. Plant Pathol..

[13] Muratali D., Derviş S., Özer G., Türkkan M., Bozoğlu T., Alkan M., Erper İ. (2025). Molecular and pathogenic characterization of *Fusarium* species associated with dry rot in stored potatoes in Kyrgyzstan. Potato Res..

[14] Christian C.L., Rosnow J., Woodhall J.W., Wharton P.S., Duellman K.M. (2025). Pathogenicity of *Fusarium* species associated with potato dry rot in the Pacific Northwest of the United States. Plant Dis..

[15] Guo J., Shi Y., Chen X., Du P., Zhao Y., Wang L. (2025). Molecular identification and pathogenicity of *Fusarium* fungi causing potato dry rot in Shanxi Province, China. J. Fungi.

[16] Pooja, Chauhan P., Kumar A., Rithesh L., Kumar A. (2025). Recent insights into potato dry rot, an emerging disease: Focusing on pathogen diversity, host–pathogen interactions, and management strategies. Microb. Pathog..

[17] Meijer B.J.M., Aissat A., Benchaalal K. (2019). Potato Storage and Processing in Algeria: Study on Potato Processing and Post-Harvest Chain in Algeria.

[18] Gutiérrez Pozo M., Verheecke Vaessen C., Kourmpetli S., Terry L.A., Medina A. (2024). Effect of temperature, relative humidity, and incubation time on the mycotoxin production by *Fusarium* spp. responsible for dry rot in potato tubers. Toxins.

[19] Zhao Y., Lv H., Li Y. (2023). Grain storage: Theory, technology and equipment. Foods.

[20] Geiser D.M., del Mar Jiménez-Gasco M., Kang S., Makalowska I., Veeraraghavan N., Ward T.J., Zhang N., Kuldau G.A., O’Donnell K. (2004). FUSARIUM-ID v. 1.0: A DNA sequence database for identifying *Fusarium*. Eur. J. Plant Pathol..

[21] Studt L., Schmidt F.J., Jahn L., Sieber C.M.K., Connolly L.R., Niehaus E.-M., Freitag M., Humpf H.-U., Tudzynski B. (2013). Two histone deacetylases, FfHda1 and FfHda2, are important for *Fusarium fujikuroi* secondary metabolism and virulence. Appl. Environ. Microbiol..

[22] Yörük E., Yli-Mattila T. (2024). Translation elongation factor 1-alpha sequencing provides reliable tool for identification of *Fusarium graminearum* species complex members. Diversity.

[23] Ngugi L.C., Abelwahab M., Abo-Zahhad M. (2021). Recent advances in image processing techniques for automated leaf pest and disease recognition: A review. Inf. Process. Agric..

[24] Bock C.H., Barbedo J.G., Del Ponte E.M., Bohnenkamp D., Mahlein A.K. (2020). From visual estimates to fully automated sensor-based measurements of plant disease severity: Status and challenges for improving accuracy. Phytopathol. Res..

[25] Orr R., Pattison A., East D., Warman N., O’Neill W., Czislowski E., Nelson P.N. (2019). Image-based quantification of Fusarium wilt severity in banana. Australas. Plant Dis. Notes.

[26] Wu L., Hwang S.-F., Strelkov S.E., Fredua-Agyeman R., Oh S.-H., Bélanger R.R., Wally O., Kim Y.-M. (2024). Pathogenicity, host resistance, and genetic diversity of *Fusarium* species under controlled conditions from soybean in Canada. J. Fungi.

[27] Gültekin M.A., Özer N., Özer G. (2025). Molecular and pathogenic characterization of *Fusarium oxysporum* f. sp. pisi isolates obtained from Turkey. Eur. J. Plant Pathol..

[28] Benhaoued F.Z., Bissati-Bouafia S., Hadjadj S., Hammoudi R. (2024). Identification and characterization of some phytopathogenic fungi in postharvest potato (*Solanum tuberosum*) in the El Oued region, Eastern Northern Sahara, Eastern Algeria. Int. J. Health Sci..

[29] Hadjebar S., Yekkour A., Djemouai N., Matmoura A., Gutierrez-Pozo M., Medina A., Meklat A., Verheecke-Vaessen C. (2024). Mycotoxin accumulation in dry rot potato tubers from Algeria and toxigenic potential of associated isolates of the *Fusarium* genus. Curr. Microbiol..

[30] Bojanowski A., Avis T.J., Pelletier S., Tweddell R.J. (2013). Management of potato dry rot. Postharvest Biol. Technol..

[31] Heltoft P., Brurberg M.B., Skogen M., Le V.H., Razzaghian J., Hermansen A. (2016). *Fusarium* spp. causing dry rot on potatoes in Norway and development of a real-time PCR method for detection of *Fusarium coeruleum*. Potato Res..

[32] Bayona L.G., Grajales A., Cárdenas M.E., Sierra R., Lozano G., Garavito M.F., de García M.C., Bernal A., Jiménez P., Restrepo S. (2011). Isolation and characterization of two strains of *Fusarium oxysporum* causing potato dry rot in *Solanum tuberosum* in Colombia. Rev. Iberoam. Micol..

[33] Harish J., Jambhulkar P.P., Bajpai R., Arya M., Babele P.K., Chaturvedi S.K., Kumar A., Lakshman D.K. (2023). Morphological characterization, pathogenicity screening, and molecular identification of *Fusarium* spp. isolates causing postflowering stalk rot in maize. Front. Microbiol..

[34] Ajmal M., Hussain A., Ali A., Chen H., Lin H. (2023). Strategies for controlling the sporulation in *Fusarium* spp.. J. Fungi.

[35] Avalos J., Estrada A.F. (2010). Regulation by light in *Fusarium*. Fungal Genet. Biol..

[36] Ezrari S., Radouane N., Tahiri A., Amiri S., Lazraq A., Lahlali R. (2021). Environmental effects of temperature and water potential on mycelial growth of *Neocosmospora solani* and *Fusarium* spp. causing dry root rot of citrus. Curr. Microbiol..

[37] Bugingo C., Infantino A., Okello P., Perez-Hernandez O., Petrović K., Turatsinze A.N., Moparthi S. (2025). From morphology to multi-omics: A new age of *Fusarium* research. Pathogens.

[38] Baturo-Ciesniewska A., Lenc L., Grabowski A., Lukanowski A. (2015). Characteristics of Polish isolates of *Fusarium sambucinum*: Molecular identification, pathogenicity, diversity and reaction to control agents. Am. J. Potato Res..

[39] Yikilmazsoy G., Tosun N. (2021). Characterization of *Fusarium sambucinum* isolates associated with potato dry rot and evaluation of cultivar susceptibility and fungicides. Turk. J. Agric. For..

[40] O’Donnell K., Ward T.J., Robert V.A.R.G., Crous P.W., Geiser D.M., Kang S. (2015). DNA sequence-based identification of *Fusarium*: Current status and future directions. Phytoparasitica.

[41] Torres-Cruz T.J., Whitaker B.K., Proctor R.H., Broders K., Laraba I., Kim H.S., Brown D.W., O’Donnell K., Estrada-Rodríguez T.L., Lee Y.H. (2022). FUSARIUM-ID v. 3.0: An updated, downloadable resource for *Fusarium* species identification. Plant Dis..

[42] Garmendia G., Umpierrez-Failache M., Ward T.J., Vero S. (2018). Development of a PCR-RFLP method based on the transcription elongation factor 1-α gene to differentiate *Fusarium graminearum* from other species within the *Fusarium graminearum* species complex. Food Microbiol..

[43] Parkland Potato Varieties Ltd. Arizona. https://www.agricopotatoes.com/en-ca/potato-varieties/arizona.

[44] Pooja, Chauhan P., Saini A.K., Kumar A., Kumar A. (2025). Use of microbial agents and botanicals for the management of potato dry rot caused by *Fusarium* species complex (FSC). J. Plant Pathol..

[45] Elsherbiny E.A., Amin B.H., Baka Z.A. (2016). Efficiency of pomegranate (*Punica granatum* L.) peels extract as a high potential natural tool towards *Fusarium* dry rot on potato tubers. Postharvest Biol. Technol..

[46] Rafiq M., Javaid A., Kanwal A., Anwar A., Khan I.H., Kanwal Q., Cheng C. (2024). GC-MS analysis and antifungal potential of flower extract of *Acacia nilotica* subsp. *indica* against *Macrophomina phaseolina*. Microb. Pathog..

[47] Rafiq M., Shoaib A., Javaid A., Perveen S., Asdullah H.U., Cheng C. (2025). Phytochemical profile of stem extract of *Carthamus oxycantha* and identification of herbicidal and antimicrobial constituents. Plant Prot. Sci..

[48] Leslie J.F., Summerell B.A. (2006). The Fusarium Laboratory Manual.

[49] Ierna A., Mauromicale G. (2024). How physicochemical and nutritional traits of potatoes may vary under field conditions over long periods. J. Sci. Food Agric..

[50] O’Donnell K., Kistler H.C., Cigelnik E., Ploetz R.C. (1998). Multiple evolutionary origins of the fungus causing Panama disease of banana: Concordant evidence from nuclear and mitochondrial gene genealogies. Proc. Natl. Acad. Sci. USA.

[51] Kumar S., Stecher G., Li M., Knyaz C., Tamura K. (2018). MEGA X: Molecular Evolutionary Genetics Analysis across computing platforms. Mol. Biol. Evol..

