# Supplementary material for: Morphological Characterization and Pathogenicity Screening of Fusarium Isolates Associated with Dry Rot of Stored Potato Tubers in Mascara, Algeria
Source: Plants (Basel). 2026 Jun 27;15(13):1999. doi: 10.3390/plants15131999 (PMC13363812; doi:10.3390/plants15131999)
Supplement: Supplementary file 1 [file plants-15-01999-s001.zip › Supplementary Figure S1.pdf]

Supplementary Figure S1: Macroscopic and microscopic observation of selected isolates in this study

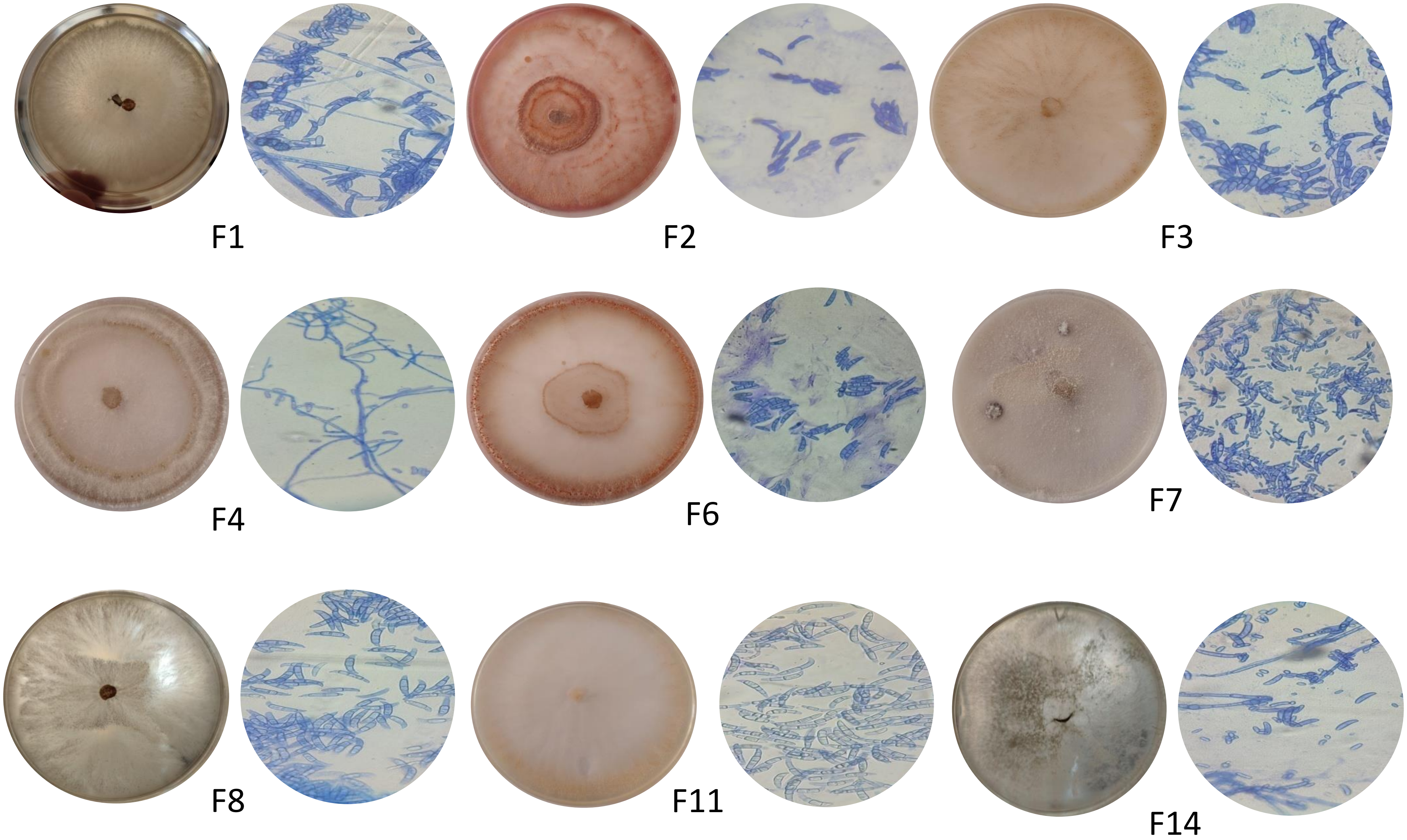

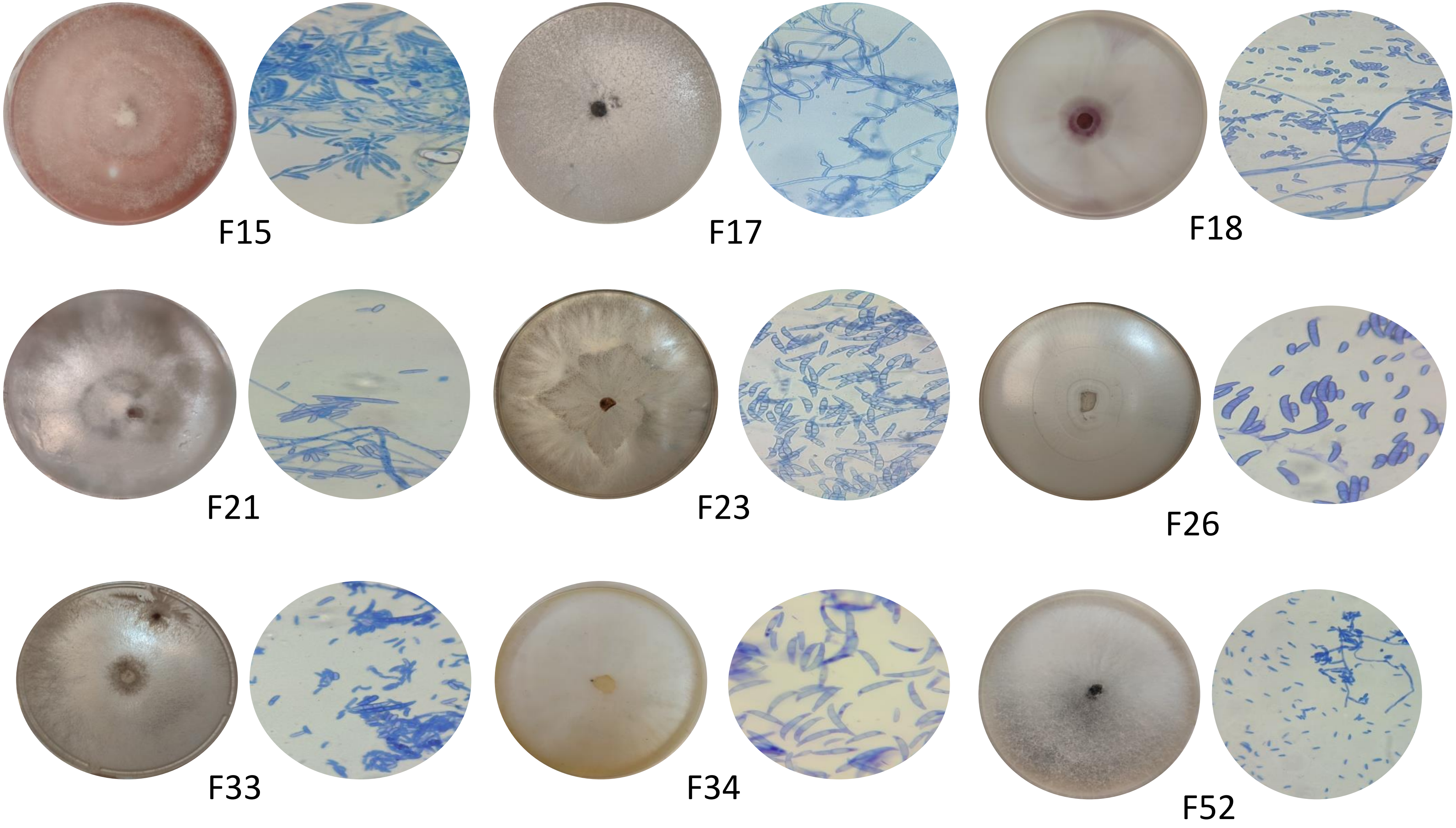
F15
F17
F18
F21
F23
F26
F33
F34
F52

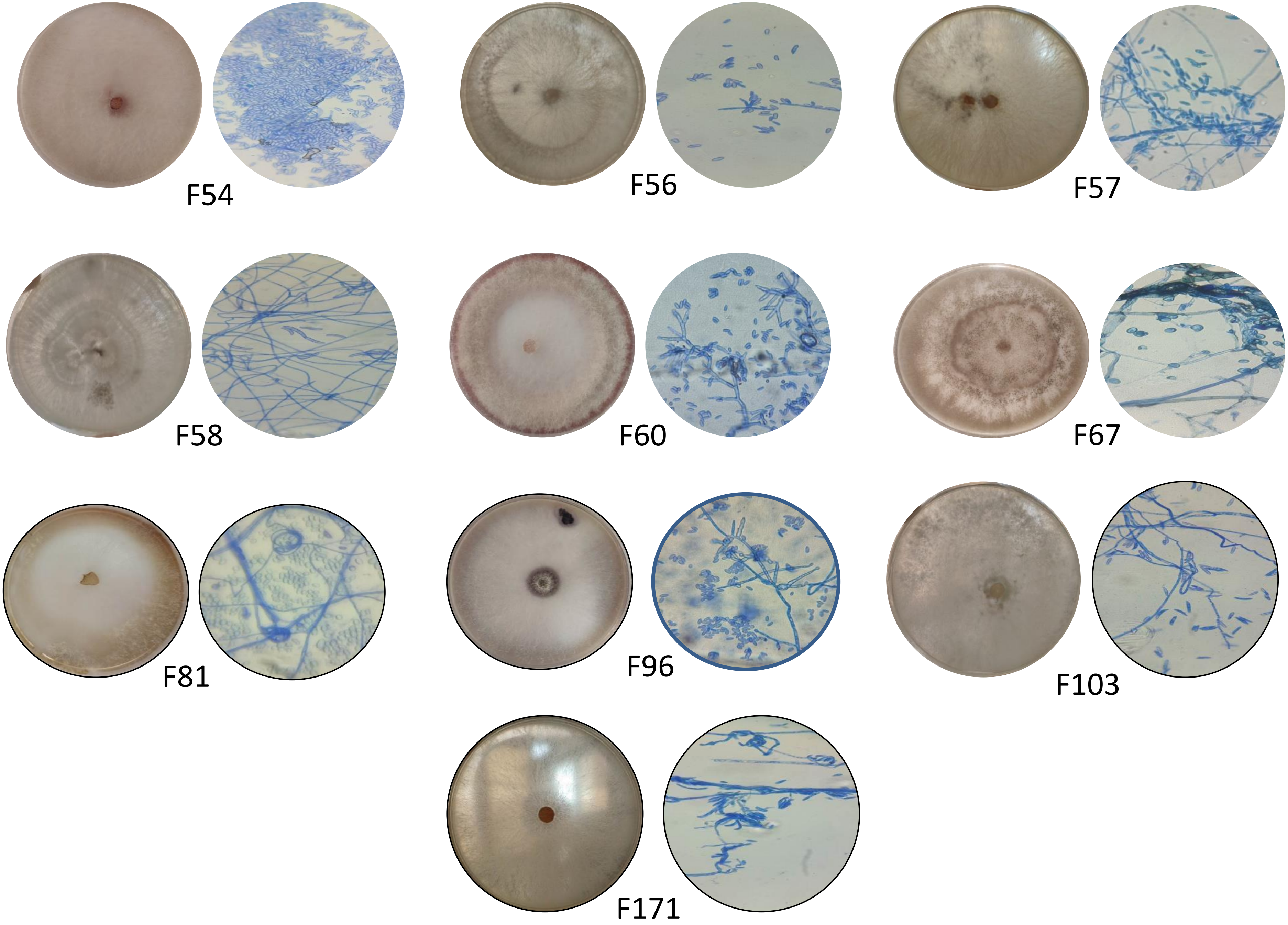
F54
F56
F57
F58
F60
F67
F81
F96
F103
F171
