# Supplementary material for: Morphological Characterization and Pathogenicity Screening of Fusarium Isolates Associated with Dry Rot of Stored Potato Tubers in Mascara, Algeria
Source: Plants (Basel). 2026 Jun 27;15(13):1999. doi: 10.3390/plants15131999 (PMC13363812; doi:10.3390/plants15131999)
Supplement: Supplementary file 1 [file plants-15-01999-s001.zip › Supplementary_Table_S1_Disease_Severity.pdf]

Supplementary Table S1. Disease-severity values of representative *Fusarium* isolates used for statistical analysis.

Isolate	Run 1 mean (%)	Run 2 mean (%)	Run 3 mean (%)	Mean (%)	SD	SE	Tukey group
F1	11.93	2.55	2.86	5.78	5.33	3.08	dc
F14	44.50	18.50	20.30	27.77	14.52	8.38	cb
F15	1.42	1.69	1.50	1.53	0.15	0.08	d
F171	53.60	59.00	46.32	52.97	6.36	3.67	a
F18	8.48	1.93	2.10	4.17	3.73	2.16	d
F2	4.95	5.80	5.35	5.37	0.43	0.25	dc
F26	1.37	1.56	1.98	1.64	0.31	0.18	d
F3	8.84	0.90	1.20	3.65	4.50	2.60	d
F34	63.50	31.68	70.00	55.06	20.51	11.84	a
F4	33.24	35.77	34.50	34.50	1.27	0.73	ab
F54	16.52	2.76	2.34	7.21	8.07	4.66	dc
F56	15.34	3.80	9.00	9.38	5.78	3.34	dc
F57	4.87	1.86	4.51	3.75	1.64	0.95	d
F67	2.36	1.57	2.21	2.05	0.42	0.24	d

Note. Values are expressed as percentage of necrotic area relative to the total tuber cross-sectional area. Run means were calculated from five tubers per isolate in each independent experimental run and used as experimental units for ANOVA. Different letters indicate significant differences among isolates according to Tukey's HSD test at $P <= 0.05$.
